# Recoverable Phospha-Michael Additions Catalyzed by a 4-*N*,*N*-Dimethylaminopyridinium Saccharinate Salt or a Fluorous Long-Chained Pyridine: Two Types of Reusable Base Catalysts

**DOI:** 10.3390/molecules26041159

**Published:** 2021-02-22

**Authors:** Eskedar Tessema, Vijayanath Elakkat, Chiao-Fan Chiu, Jing-Hung Zheng, Ka Long Chan, Chia-Rui Shen, Peng Zhang, Norman Lu

**Affiliations:** 1 Institute of Organic and Polymeric Materials, National Taipei University of Technology, Taipei 106, Taiwan; eskedartessema18@gmail.com (E.T.); vijayanathe123@gmail.com (V.E.); st121321@gmail.com (J.-H.Z.); ka-longabc@gmail.com (K.L.C.); 2 Department of Pediatrics, Linkou Medical Center, Chang Gung Memorial Hospital, Taoyuan 333, Taiwan; 3 Graduate Institute of Clinical Medical Sciences, College of Medicine, Chang Gung University, Taoyuan 333, Taiwan; 4 Department of Medical Biotechnology and Laboratory Sciences, College of Medicine, Chang Gung University, Taoyuan 333, Taiwan; crshen@mail.cgu.edu.tw; 5 Department of Ophthalmology, Linkou Medical Center, Chang Gung Memorial Hospital, Taoyuan 333, Taiwan; 6 Department of Chemistry, University of Cincinnati, Cincinnati, OH 45221-0172, USA; 7 Development Center for Smart Textile, National Taipei University of Technology, Taipei 106, Taiwan

**Keywords:** phospha-Michael, recoverable, fluorous, DMAP, long-chained, pyridine, catalysis, adduct, thermomorphic, phosphite

## Abstract

Phospha-Michael addition, which is the addition reaction of a phosphorus-based nucleophile to an acceptor-substituted unsaturated bond, certainly represents one of the most versatile and powerful tools for the formation of P-C bonds, since many different electrophiles and P nucleophiles can be combined with each other. This offers the possibility to access many diversely functionalized products. In this work, two kinds of basic pyridine-based organo-catalysts were used to efficiently catalyze phospha-Michael addition reactions, the 4-*N*,*N*-dimethylaminopyridinium saccharinate (DMAP·Hsac) salt and a fluorous long-chained pyridine (4-*R*_f_-CH_2_OCH_2_-py, where *R*_f_ = C_11_F_23_). These catalysts have been synthesized and characterized by Lu’s group. The phospha-Michael addition of diisopropyl, dimethyl or triethyl phosphites to α, β-unsaturated malonates in the presence of those catalysts showed very good reactivity with high yield at 80–100 °C in 1–4.5 h with high catalytic recovery and reusability. With regard to significant catalytic recovery, sometimes more than eight cycles were observed for DMAP·Hsac adduct by using non-polar solvents (e.g., ether) to precipitate out the catalyst. In the case of the fluorous long-chained pyridine, the thermomorphic method was used to efficiently recover the catalyst for eight cycles in all the reactions. Thus, the easy separation of the catalysts from the products revealed the outstanding efficacy of our systems. To our knowledge, these are good examples of the application of recoverable organo-catalysts to the DMAP·Hsac adduct by using non-polar solvent and a fluorous long-chained pyridine under the thermomorphic mode in phospha-Michael addition reactions.

## 1. Introduction

The chemistry of phosphonates has inspired an increasing interest following their synthetic and biological reputation. As a result, the development of new methodologies for their preparation is an important goal in organic synthesis [1,2,3,4]. To date, intensive studies have been reported in their synthetic application in biomedical fields, such as peptide mimicking [5], enzyme inhibition [6], metabolic inquiries [7], pharmacological activity study [8] and biological activities [9]. These transformations, however, require the use of a base catalyst to promote tautomeric equilibria of H-phosphonates in favor of a reactive form of phosphites [10].

Among the main methods of the synthesis of phosphonates through C-P bond formation is the addition of phosphite nucleophile across the carbon–carbon double bond, which is called the phospha-Michael reaction [11,12]. This reaction has been catalyzed by metal oxides [13], acids [14], bases [15,16], radical initiator [17,18] transition metal catalysts [19], tetramethylguanidine [20], microwave [21], HClO_4_.-SiO_2_ [22], nano-sized ZnO [23], sodium stearate [24], and so on. Although phospha-Michael addition could proceed by these catalyzed methods, many of these reagents cannot be reused, and in many instances, long reaction time; drastic reaction conditions; and sometimes, according to the nature of the catalyst, tedious work-up is needed. Therefore, the development of a new method to overcome these shortcomings still remains an ongoing challenge for the synthesis of these significant scaffolds. Recently, some recoverable catalyst systems have also been reported to be active, such as magnetically recyclable heterogeneous organic base [25] and 3-aminopropylated silica gel [26].

Reported here, the 4-*N*,*N*-dimethylaminopyridinium saccharinate (DMAP·Hsac) salt was used as a catalyst in the current work. This salt was synthesized by the reversible acid-base reaction of the basic 4-*N*,*N*-dimethylaminopyridine (DMAP) with the acidic saccharine (Hsac). During the equilibrium, there is always a very small amount of free DMAP present in the solution (see Equation 1), and this free DMAP can homogeneously and effectively catalyze the phospha-Michael addition reactions [27]. Additionally, this salt of 4-*N*,*N*-dimethylaminopyridinium saccharinate (DMAP·Hsac), which is an ionic compound, is usually insoluble in most non-polar solvents. It can be easily precipitated out to achieve the phase separation by adding the non-polar solvents. (e.g., ether, hexane etc.) [27,28]. This special solubility property of DMAP·Hsac makes the adduct act as a good catalytic bridge between heterogeneous and homogeneous catalysis, as the addition of non-polar solvent makes the catalyst precipitate out. Likewise, in the 2nd part of this research, the fluorous long-chained pyridine (4-*R*_f_-CH_2_OCH_2_-py where *R*_f_ = C_11_F_23_), as a base catalyst, works under thermomorphic conditions with a feature of homogeneously catalyzing phospha-Michael addition at a high temperature in a neat reaction where the reactants serve as the solvent, forming heterogeneous precipitation at a lower temperature. The thermomorphic method is an alternative way to use the different solubility of fluorous long-chained compounds between high and low temperature in the common organic compounds [29,30,31,32,33,34,35]. In this method, a fluorous chain-containing pyridine-based catalyst could be soluble at high temperatures during the catalytic reactions, but insoluble at room temperature for the recovery of catalysts via a simple liquid–solid separation [36,37,38].
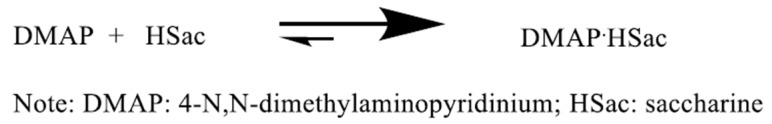
(1)

In this work, two kinds of basic pyridine-based organo-catalysts, which are the DMAP·Hsac adduct and a fluorous long-chained pyridine (py), were used to efficiently catalyze phospha-Michael addition reactions. To our knowledge, these are two good examples of recoverable phospha-Michael addition reaction catalyzed by organo-bases, which are DMAP·Hsac adduct in neat condition and a fluorous long-chained py under thermomorphic mode. Taking into consideration their good catalytic efficiency and good heterogenous separation, these two catalysts could be regarded as very good alternatives to most homogeneous catalysts.

## 2. Results and Discussion

### 2.1. Recoverable DMAP·HSac-Catalyzed Phospha-Michael Addition Reaction

#### 2.1.1. Synthesis of DMAP·HSac (Catalyst **A**)

**Scheme 1:**
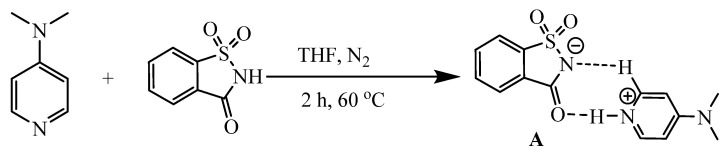
Synthesis of the DMAP·HSac adduct (**A**).

The preparation of DMAP·HSac (**A**) followed Lu’s literature procedure [28]. The reaction of DMAP with saccharin, as shown in Scheme 1, in tetrahydrofuran (THF) solvent at 60 °C, took place for 2 h in a nitrogen atmosphere to yield a white solid product of the adduct (**A**).

#### 2.1.2. Recoverable DMAP·HSac-Catalyzed Phospha-Michael Addition Reaction

**Scheme 2:**
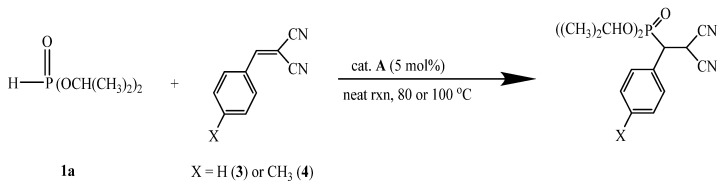
The DMAP·HSac (**A**)-catalyzed phospha-Michael addition of diisopropyl phosphite (**1a**) with benzylidenemalononitrile-type substrates [3 (X = H); 4 (X = CH_3_)].

**Scheme 3:**
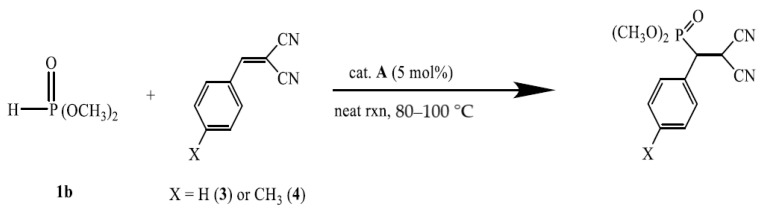
**A**-catalyzed phospha-Michael addition of dimethyl phosphite (**1b**) with 2-benzylidenemalononitrile-type substrates [**3** (X = H); **4** (X = CH_3_)].

The DMAP·HSac adduct **A** was then examined for the following neat phospha-Michel addition reactions, where **A** catalyzed addition of alkyl (R group) substituted diorganophosphite compounds [**1a** (R= isopropyl); **1b** (R= methyl)] and tris(organo)phosphite compound [**2** (R = ethyl)] with 2-benzylidinemelanonitrile-type substrates [**3** (X = H); **4** (X = CH_3_)] as indicated in Scheme 2 and Scheme 3.

*a.*  ***A**-catalyzed phospha-Michael addition of diisopropyl phosphite (**1a**) and benzylidenemalononitrile (**3**)*

As shown in Scheme 2, the DMAP·HSac (**A**)-catalyzed phospha-Michael addition reaction of diisopropyl phosphite (**1a**) with 2-benzylidenemalononitrile (**3**) was selected to demonstrate the feasibility of recycling usage with **A** as a catalyst using a solvent free method, at 80 °C mainly for 1 h. In this time, the reaction was successfully carried out to afford the product in quantitative conversions, as shown in Scheme 2 and Table 1. At the end of each cycle, the product mixtures were cooled to room temperature, and non-polar solvent (e.g., ether) was added to the reaction tube and centrifuged, and the catalyst was recovered by decantation. The recovered catalyst **A** was dried under vacuum and proceeded to the next cycle. The products were quantified with GC/MS analysis by comparison to internal standard (anisole). As shown in Table 1, **A**-catalyzed phospha-Michael addition of **1a** with **3** could give rise to good yield and recycling results for a total of eight times without using any solvent.

The phospha-Michel addition reaction of **1a** with **3** showed high yield and good recycling result for eight cycles by using 5 mol% catalyst at 80 °C. By further lowering the catalytic loading to 1 mol% at 100 °C, a higher yield was still obtained within 1 h (see Table 2). Additionally, catalyst **A**, which is thermally robust and is stable even when temperature is higher than 100 °C, was effectively recycled for the increased number of cycles (16 cycles) almost without a loss in catalytic activity. The average yield for all the consecutive runs was 99%, which clearly demonstrates the practical reusability of this catalyst. Thus, it can be said that catalyst **A** showed a robust catalytic activity with a TON = *ca* 100 and a TOF = *ca* 100 and also with excellent recovery and good thermal stability.

*b.*  ***A**-catalyzed phospha-Michael addition of diisopropyl phosphite (**1a**) with 2-(4-methylbenzylidene)malononitrile (**4**)*

The catalyst **A** -catalyzed phospha-Michael addition reaction of diisopropyl phosphite (**1a**) with 2-(4-methylbenzylide)nemalononitrile (**4)** proceeded with a good yield at 80 °C in 2.5–3.5 h. However, the introduction of the electron donating group (X = CH_3_) into the benzene ring of compound **4** reduced the rate of the reaction, leading to increased reaction time (Table 3) compared to the unsubstituted one (**3**, X = H).

*c.*  ***A**-catalyzed phospha-Michel addition of dimethyl phosphite (**1b**) and 2-benzylidenemalononitrile (**3**)*

Table 4 shows the result of the reactivity and recycling of the **A**-catalyzed phospha-Michael addition reaction of dimethyl phosphite (**1b**) with 2-benzylidenemalononitrile (**3**). The results of Table 1 show a slightly shorter reaction time at later stages of recycling than those of Table 4. The change of the isopropyl group to methyl group on the phosphite substrate (see Scheme 2 and Scheme 3) did not show much change in the rate of the reaction. Thus, this change does not have a significant effect on the nucleophilicity of the phosphite substrate.

*d.*  ***A**-catalyzed phospha-Michael addition of dimethyl phosphite (**1b**) and 2-(4-methylbenzylidene)malononitrile (**4**)*

In Table 5, the recycling results of the **A**-catalyzed phospha-Michael addition of dimethyl phosphite (**1b**) with 2-(4-methylbenzylidene)malononitrile (**4**) are presented. This reaction proceeded with good yield, mainly for 2 h at 80–100 °C. However, the introduction of the electron donating group (X = CH_3_) into the malononitrile substrate (**4,** X = CH_3_) reduced the rate of the reaction, leading to an increased reaction time and temperature (Table 5) compared to the unsubstituted substrate (**3**, X = H).

*e.*  ***A**-catalyzed phospha-Michael addition of triethyl phosphite (**2**) and 2-benzylidenemalononitrile (**3**)*

**Scheme 4:**
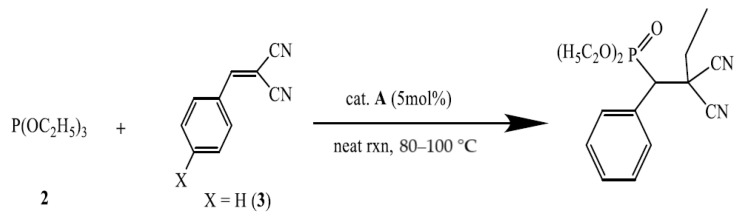
**A**-catalyzed phospha-Michael addition of triethyl phosphite (**2**) with 2-benzylidenemalononitrile (**3**).

As shown in Table 6, the **A**-catalyzed phospha-Michael addition of triethyl phosphite (**2**) with 2-benzylidenemalononitrile (**3**) demonstrated good product yield and catalytic recovery in 1 h for the first four cycles. However, it was found that the catalytic performance of the recovered cat. **A** slightly diminished after each step to the 8^th^ cycle. In Scheme 4, the different kind of phosphite (**2**) used in this reaction is seen to yield an alkyl substituted product.

In summary, in the **A**-catalyzed phospha-Michel addition of phosphite reagents (**1a, 1b** or **2**) with 2-benzylidenemalononitrile-type substrates (**3** or **4**), the methyl, ethyl and isopropyl substituents of the phosphite did not show a significant difference in their yields and reaction times. However, after several recycles, the triethyl substituted phosphite did not maintain its reaction speed as the previous cycles (see Table 6). Additionally, for the phosphorus-acceptor saturated bond containing 2-benzylidenemalononitrile-type substrates (**3** or **4**), the unsubstituted compound **3** showed a shorter reaction time than the CH_3_ substituted compound **4**. Here, the electron releasing CH_3_ group was found to retard the speed of the reaction. Hosseini-Sarvari and Etemad reported a nanosized zinc oxide catalyzed similar reaction by using [P(O)(OEt)_2_] phosphite substrate showing a similar trend of shorter reaction time for the unsubstituted malononitrile substrate (2.5 h) than the methyl substituent (5 h) [23,25].

For the base **A**-catalyzed reactions of diorganophosphite compounds (**1a** or **1b**) with 2-benzylidenemalononitrile-type substrates (**3** or **4**), the reactions followed the cited reaction mechanism [39,40], demonestrating the base assisted P-H bond cleavage and adding the H and P-containing moiety into the two sides of the double bond (see Figure S1 in SM). However, the **A**-catalyzed similar reaction of tris(organo)phosphite (**2**) with 2-benzylidenemalononitrile (**3**) followed a different mechanism through the O-C bond cleavage of one -OC_2_H_5_ group to add the phosphonate group [P(O)(O C_2_H_5_)_2_] and the ethyl groups into the two sides of the double bond (Scheme 4).

#### 2.1.3. Kinetic Study of **A**-Catalyzed Phospha-Michael Addition Reaction of Diisopropyl Phosphite (**1a**) with Benzylidenemalononitrile (**3**)

**Figure 1:**
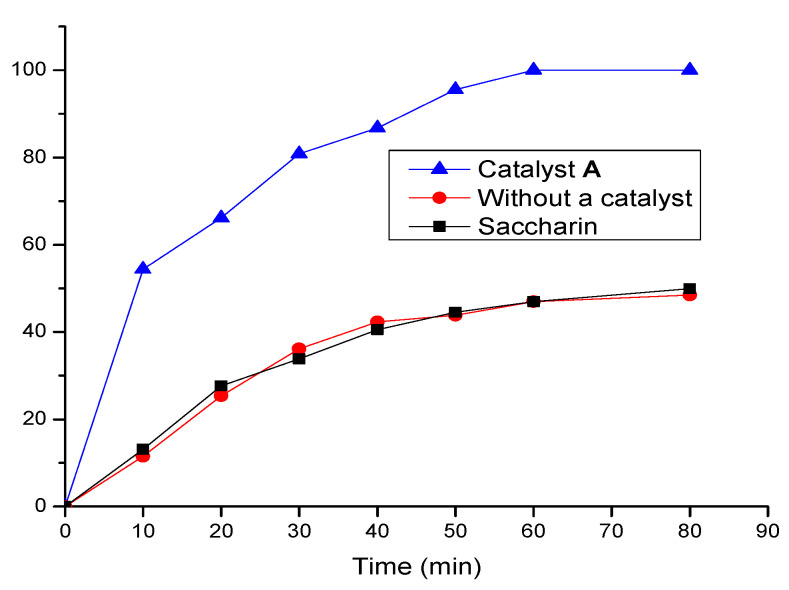
Kinetic study of phospha-Michael addition of diisopropyl phosphite (**1a**) with benzylidenemalononitrile (**3**) with and without a catalyst **A**. (Note: saccharin as a catalyst was also tested, but it showed no catalytic effect.)

The phospha-Michael addition of diisopropyl phosphite (**1a**) with benzylidenemalononitrile (**3**) was also studied kinetically at 80 °C (see Figure 1). The reaction showed a drastic increase in product concentration within the initial 10 min to 54 %, and the reaction reached 100 % yield after 1 h. The integrated rate law derived from the concentration of reactant **3** vs. time showed the ln[reactant **3**] = −kt + ln[reactant **3**]_o_ plot with a rate constant k = 0.057 and R^2^ = 0.97, where [reactant **3**]_o_ = 3 M and ln[reactant **3**]_o_ = 1.074, as shown in Figure S2 (in supplementary material). This kinetically monitored reaction showed the turnover frequency (TOF) to be 19.8 h^−1^ at 1 h. However, for the same reaction, only about 50% product yield was found after 90 min without using catalyst **A** or by using saccharine as a catalyst (see in Figure 1). Likewise, Sobani and her coworkers reported the reaction of diethyl phosphite with 2-benzylidenemalononitrile (**3**) in the absence of the catalyst to give a yield of 60% in 24 h. The reactions without a catalyst led to the formation of the desired product in low yields after a long reaction time [25]. Furthermore, Sarvari and coworkers reported that the reaction of diethyl phosphite with the electron withdrawing group substituted 2-[(4-chlorophenyl) methylene]malononitrile in the absence of the catalyst gave no product after 24 h [23]. Overall, these control experiments showed the same trend, which indicated that without a catalyst, the phospha-Michael addition reactions were either very slow or showed no reaction.

### 2.2. Recoverable Fluorous Long-Chained Pyridine-Catalyzed Phospha-Michael Addition Reaction

#### 2.2.1. Synthesis of *R*_f_CH_2_OCH_2_-py (Catalyst **B**)

**Scheme 5:**
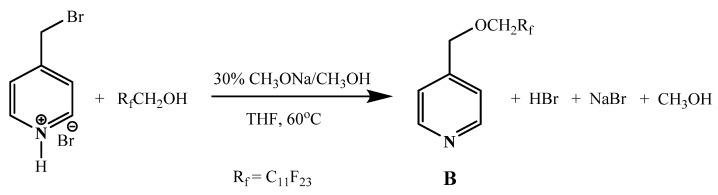
Synthesis of *R*_f_CH_2_OCH_2_-py catalyst **B**.

The synthesis of *R*_f_CH_2_OCH_2_-py (catalyst **B**) proceeds through two steps, as reported in the literature [41]. The initial step starts from the deprotonation of readily available fluorinated alcohols. Thus, *R*_f_-CH_2_OH, where *R*_f_ = C_11_F_23_, was treated with 30% CH_3_ONa/CH_3_OH to give the corresponding alkoxides whose nucleophilic attack on 4-(BrCH_2_)-py·HBr gave rise to the synthesis of fluorous long-chained pyridine **B**, [4-(*R*_f_CH_2_OCH_2_)-py)] (see Scheme 5).

#### 2.2.2. Recoverable Fluorous *R*_f_-py-Catalyzed Phospha-Michael Addition

*a.*  ***B**-catalyzed phospha-Michael addition of diisopropyl phosphite (**1a**) with 2-benzylidenemalononitrile (**3**)*.

**Scheme 6:**
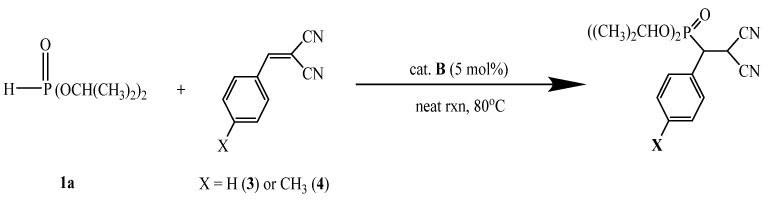
The **B**-catalyzed phospha-Michael addition of diisopropyl phosphite (**1a**) with 2-benzylidenemalononitrile-type substrates [**3** (X = H), or **4** (X = CH_3_)].

In Scheme 6, the **B**-catalyzed phospha-Michael addition of diisopropyl phosphite (**1a**) with 2-benzylidenemalononitrile (**3**) was selected to demonstrate the feasibility of recycling usage with **B** as a catalyst using a solvent free method, at 80 °C for 1 h. At the end of each cycle, the product mixtures were cooled to room temperature, resulting in catalyst precipitation due to their thermomorphic behavior where the solubility of the catalyst increased dramatically with the increasing temperature [36,38]. Finally, after centrifugation, the catalyst was recovered by decantation. The recovered catalyst **B** was dried under vacuum and proceeded to the next cycle. The products were quantified with GC/MS analysis through comparison to the internal standard (e.g., anisole). As shown in Table 7, the **B**-catalyzed phospha-Michael addition reaction of **1a** with **3** could give rise to good recycling results for a total of eight times without using any solvent.

*b.*  ***B**-catalyzed phospha-Michael addition diisopropyl phosphite (**1a**) with 2-(4-methylbenzylidene)malononitrile (**4**)*

The **B**-catalyzed phospha-Michael addition of diisopropyl phosphite (**1a**) with 2-(4-methylbenzylidene)malononitrile (**4**) was successfully carried out under thermomorphic conditions at 5 mol% catalyst loading as shown in Scheme 6 above (Table 8). However, an elongated reaction time was observed compared to the reaction using unsubstituted 2-benzylidenemalononitrile (**3**). It followed the same trend compared to the similar **A**-catalyzed reaction mentioned above (see Table 3).

*c.*  ***B**-catalyzed phospha-Michael addition of dimethyl phosphite (**1b**) with 2-benzylidenemalononitrile (**3**)*

**Scheme 7:**
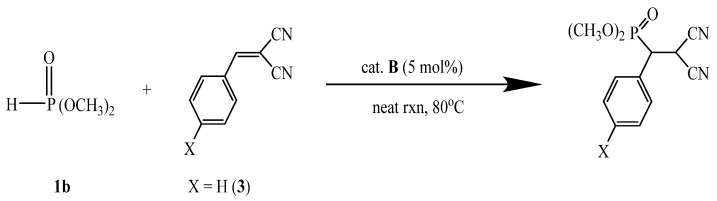
**B**-catalyzed phospha-Michael addition of dimethyl phosphite (**1b**) with 2-benzylidenemalononitrile (**3**).

The **B**-catalyzed phospha-Michael addition of dimethyl phosphite (**1b**) with 2-benzylidenemalononitrile (**3**) (see Scheme 7) was also found to be effective with good recoveries, as shown in Table 9. The reaction time recorded for this reaction is almost the same as the time for **A**-catalyzed similar reaction.

In summary, in the **B**-catalyzed phospha-Michael addition of phosphite reagents (**1a** or **1b**) with 2-benzylidenemalononitrile-type substrates (**3** or **4**), no significant difference was recorded for the methyl or the isopropyl substituents of the phosphite as reported for the **A**-catalyzed reactions above. However, the **B**-catalyzed reaction of the unsubstituted compound **3** showed a better reaction time than the CH_3_ substituted compound **4.** The outcomes from the **B**-catalyzed reactions are comparable to those of the **A**-catalyzed reactions, which could still be explained by the electronic effect of the electron releasing property of the CH_3_ group.

#### 2.2.3. Kinetic Study of **B**-Catalyzed Phospha-Michael Addition of Diisopropyl Phosphite (**1a**) with Benzylidenemalononitrile (**3**)

The **B**-catalyzed phospha-Michael addition reaction of diisopropyl phosphite (**1a**) with benzylidenemalononitrile (**3**) showed an increase in product concentration to 49 % in the initial 10 min. Then, 100 % of reactant **3** was converted to a product after 1 h, as shown in Figure S3 in the supplementary material. The integrated rate law derived from the concentration of reactant **3** vs. time showed ln[reactant **3**] = −kt + ln[reactant **3**]_o_ plot with a rate constant k = 0.055 and R^2^ = 0.95, where ln[reactant **3**]_o_ = 1.154, as shown in Figure S4 in the supplementary material. This kinetically monitored reaction showed the turnover frequency (TOF) to be 19.2 h^−1^ at 1 h. When comparing the reaction rate of catalyst **B** with that of catalyst **A,** both of the reactions were pseudo first order reactions with catalyst **A**, which had a higher rate constant (k = 0.057) than that (k = 0.055) of catalyst **B**. The presence of an electron withdrawing fluorous chain on the pyridyl nitrogen had an electronic effect on the activity of the catalyst. Thus, catalyst **B** showed a slight decrease in reaction rate when compared to catalyst **A**.

## 3. Experimental

### 3.1. General Procedure

General Procedures HP 6890 GC containing a 30 m 0.250 mm HP-1 capillary column with a 0.25 mm stationary phase film thickness was used to censor the reaction. The same GC instrument with a 5973 series mass selective detector was used to Acquire GC/MS data. The flow rate was 1 mL/min and splitless. Infra-red spectra were obtained on a Perkin Elmer RX I FT-IR Spectrometer. NMR spectra were recorded on Bruker AM 500 and Joel AM 200 using 5 mm sample tubes. The CDCl_3_, deuterated DMF and deuterated DMSO were the references for both ^1^H and ^13^C NMR spectra, and Freon^®^ 11 (CFCl_3_) was the reference for ^19^F NMR spectra.

### 3.2. Starting Materials

The employed chemicals, reagents and solvents were commercially available and used as received. Diisopropyl phosphite, dimethyl phosphite, triethyl phosphite, 2-benzylidenemalononitrile, 2-(4-methylbenzylidene)malononitrile (note: the local chemical vendors in Taiwan could only supply us with these substrates, which include three phosphites and two malononitriles.), DMAP, saccharin, C_11_F_23_CH_2_OH and 30% CH_3_ONa/CH_3_OH were purchased from either Aldrich or SynQuest.

### 3.3. Preparation of Catalyst **A**

DMAP (0.5 g, 4.09 mmol) and saccharin (0.74 g, 4.09 mmol) were transferred to a 250 mL round bottomed flask. An amount of 100 mL of anhydrous THF was added to the mixture and refluxed at 60 °C overnight. The initial stage the reaction mixture appeared to be a white turbid solution. The reaction mixture turned transparent by the end of the reaction. THF was removed by applying the vacuum to obtain the white solid precipitate of DMAP·HSac adduct with 95% yield (see Scheme 1).

#### Analytical Data of Catalyst **A**

Catalyst A has been prepared by Lu’s group [28] before as a recyclable acylation catalyst. Thus, the FT-IR and NMR spectra of catalyst **A** were used to identify the compound (see FT-IR spectrum in Section 5.1 and Figure S5 in SM).

### 3.4. Preparation of Catalyst **B**

*R*_f_CH_2_OH (11.0 mmol) and CH_3_ONa (30% in methanol) (10.0 mmol) were charged into a N_2_ filled two neck round-bottomed flask, then stirred continuously at 65 °C for 4 h before methanol was vacuum pumped to facilitate the reaction conversion to the fluorinated alkoxide product. The fine powdered fluorinated alkoxide (10.0 mmol) was then dissolved in dry THF (30 mL), and (BrCH_2_)-py·HBr (10 mmol) was added. The mixture was allowed to reflux for 4 h, and the completeness of the reaction was checked by sampling the reaction mixtures and analyzing the aliquots with GC/MS. Finally, the pure product was isolated by using CH_2_Cl_2_/H_2_O extraction to find a white solid material with 78 % yield (see Scheme 5) [42,43,44,45].

#### Analytical Data of Catalyst **B** (4-23F-py)

^1^H-NMR (500 MHz, DMSO-*d_6_*, at 80 °C): δ (ppm) 8.55 (d, *J* = 6 Hz, 2H, H2, H5), 7.30 (d, *J* = 6 Hz, 2H, H3, H6) 4.74 (s, 2H, py-CH_2_), 4.23 (t, *J* = 15 Hz, 2H, CH_2_CF_2_), ^19^F NMR (470.5 MHz, d-DMSO, at 80 °C) δ −80.9 (t, *^3^J_FF_* = 7.52, 3F, -CF_3_), −119.3 (2F), −121.2 (12F), −122.2 (2F), −122.8 (2F), −125.6 (2F); ^13^C NMR (113 MHz, d-DMSO, at 80 °C) δ 72.5 (py-CH_2_), 68.2 (CH_2_CF_2_), 122.5, 147.0, 150.5, (py, 5 Cs), 105.0~116.0 (C_11_F_23_). FT-IR (cm^−1^): v (py, m) 1603.8, 1468.1; v (CF_2_ stretch, s) 1200.6, 1146.3. MS (M^+^; *m*/*z* =): 691 (M^+^), 122 (C_7_H_8_NO), 108 (C_6_H_6_NO), 92(C_6_H_6_N) (see FT-IR spectrum in Section 5.2 and Figure S6 in SM).

### 3.5. Procedures in Catalytic Phospha-Michael Addition Reaction

The reactants 2-benzylidenemalononitrile substrate (115 mg, 1 equivalent), phosphite reagent (2 equivalent) and the catalyst (**A** or **B**, (0.05 equivalent)) were added together, and the reaction was carried out without solvent for all the catalysts (see Scheme 2, Scheme 3, Scheme 6 and Scheme 7). Then, the reaction mixture was set to react at 80–100 °C for the given period of time before GC/MS analysis was performed to confirm the completion of the reaction.

### 3.6. Procedures in Catalytic Recovery of DMAP·HSac (**A**)

In a typical run, 2-benzylidenemalononitrile substrate (115 mg, 0.75 mmol) and phosphite reagent (1.5 mmol) were added into a 10 mL reaction tube containing a magnetic stirrer bar, then the DMAP·HSac (A) (5 mol%) was added to the reaction tube. The reaction was carried out under solvent free conditions. Then, the reaction mixture was set to react at 80–100 °C under N_2_ gas for the given period of time. After the completion of the reaction, the product mixtures were cooled down to room temperature (25 °C), and then 5 mL of non-polar solvent (e.g., diethyl ether) was added to the reaction mixture. The reaction tube was centrifuged to separate the catalyst and reaction mixture. After the catalyst was recovered by decantation and washed three times by the same solvent, it was then dried under vacuum, and the same amounts of reactants were used to carry out the next cycle of reaction. Once the catalytic recovery was complete, GC/MS analysis was performed, the pure product was isolated by using CH_2_Cl_2_/H_2_O extraction and the CH_2_Cl_2_ layer was pumped under reduced pressure to obtain the pure product. The product was finally analyzed by using ^1^H NMR spectroscopy.

### 3.7. Procedures in Catalytic Recovery of R_f_-py Catalyst (**B**)

For the *R*_f_-py catalyst, 2-benzylidenemalononitrile substrate (115 mg, 0.75 mmol) and phosphite reagent (1.5 mmol) were added into a 10 mL reaction tube containing a magnetic stirrer bar, then catalyst **B** (5 mol%) was added. The reaction was carried out under solvent free conditions. The excess phosphite in the reaction acted as solvent. The catalyst showed thermomorphic properties at 80 °C. The reaction mixture was set to react at 80–100 °C under N_2_ gas for the given period of time. After the completion of reaction, the product mixtures were cooled down to room temperature (25 °C) to let the catalyst getting precipitated at the bottom of the reaction tube. After centrifugation, the supernatant solution containing the reaction mixture was removed carefully by using a syringe. The precipitated catalyst **B** was washed three times by the same solvent (e.g., ether) and dried under vacuum. After GC/MS analysis was complete, the product was isolated using CH_2_Cl_2_/H_2_O extraction and analyzed by using ^1^H NMR spectroscopy and the GC/MS method.

## 4. Conclusions

Developing a recoverable catalyst with a better activity, easier separation and effective recycling with a better yield is the goal of scientists. Reported here, this work showed effective catalysis by using pyridine-based organo-catalyst bases, the DMAP·Hsac adduct and a fluorous long-chained pyridine. In the **A**-catalyzed phospha-Michael addition of phosphite reagents with 2-benzylidenemalononitrile-type substrates, the methyl, ethyl and the isopropyl substituents of the phosphite did not show a significant difference in their yields and reaction times. However, after several cycles, the triethyl substituted phosphite did not maintain its reaction speed. Additionally, for the unsaturated malonates substrates, the unsubstituted compound showed a shorter reaction time than the CH_3_ substituted compound. Here, the electron releasing CH_3_ group was found to retard the speed of the reaction. Generally, it was found that the DMAP·Hsac (**A**) catalyst system showed a relatively better catalytic activity than its fluorous long-chained pyridine (**B**) counterpart. This could result from the effect of the strong electron-withdrawing property of the fluorous chain on the pyridyl nitrogen, so pyridyl nitrogen basicity was reduced. In these catalyzed reactions, solubility differences and thermomorphic properties were effectively used to recover the DMAP·Hsac adduct in Section 2.1 and a fluorous long-chained pyridine in Section 2.2, respectively. The product yield was also found to be high in every reaction run; sometimes, it even reached 100%. In summary, these catalyst systems were proven to be successful with a very high yield and are very effectively recycled, sometimes up to 16 times (as shown in Table 2), almost without a loss of activity. Both of the recoverable py-based systems reported here are supposed to also work effectively for any kind of recoverable phospha-Michael addition and most recyclable base-catalyzed reactions in general—e.g., recyclable base-catalyzed aldol condensation and recyclable base-catalyzed racemization. Therefore, we believe that these two types of organo-catalysts with heterogenous separation may also eliminate the involvement of metal-based catalyst systems in these kinds of catalytic reactions in the near future.

## Figures and Tables

**Table 1:** Recycling results of **A**-catalyzed phospha-Michael addition of diisopropyl phosphite (**1a**) with benzylidenemalononitrile (**3**) at 80 °C.

Cycle No.	Time (h)	Temp. (°C)	Yield (%) ^a^	TON
**1**	1	80	99 (97) ^b^	19.8
**2**	1	80	99	19.8
**3**	1	80	99 (96)	19.8
**4**	1	80	99	19.8
**5**	1	80	99 (95)	19.8
**6**	1	80	98	19.6
**7**	1.5	80	99	19.8
**8**	1.5	80	99	19.8

Reaction conditions: temp (T) = 80 °C, cat. **A** (5 mol%), **1a** (0.25 mL, 1.5 mmol), **3** (115 mg, 0.75 mmol), solvent free reaction. ^a^ Measured by GC/MS; ^b^ isolated yield in parenthesis.

**Table 2:** Recycling results of **A**-catalyzed phospha-Michael addition of diisopropyl phosphite (**1a**) with benzylidenemalononitrile (**3**) at 100 °C.

Cycle No.	Time (h)	Temp. (°C)	Yield (%) ^a^	TON
**1**	1	100	100 (98) ^b^	100
**2**	1	100	100	100
**3**	1	100	99 (97)	99
**4**	1	100	100	100
**5**	1	100	99 (98)	99
**6**	1	100	100	100
**7**	1	100	100	100
**8**	1	100	100	100
**9**	1	100	99	99
**10**	1	100	100	100
**11**	1	100	100	100
**12**	1	100	100	100
**13**	1	100	100	100
**14**	1	100	100	100
**15**	1	100	100	100
**16**	1	100	100	100

Reaction conditions: T = 100 °C, cat. **A** (1 mol%), **1a** (0.25 mL, 1.5 mmol), **3** (115 mg, 0.75 mmol), solvent free reaction. ^a^ Measured by GC/MS; ^b^ isolated yield in parenthesis.

**Table 3:** Recycling results of **A**-catalyzed phospha-Michael addition of diisopropyl phosphite (**1a**) with 2-(4-methylbenzylidene)malononitrile (**4**).

Cycle No.	Time (h)	Temp. (°C)	Yield (%) ^a^	TON
**1**	2.5	80	99 (99) ^b^	19.8
**2**	2.5	80	97	19.4
**3**	3	80	99 (96)	19.8
**4**	3	80	99	19.8
**5**	3	80	96 (95)	19.2
**6**	3	80	96	19.2
**7**	3	80	95	19
**8**	3.5	80	97	19.4

Reaction conditions: T = 80 °C, cat. **A** (5 mol%), **1a** (0.23 mL, 1.37 mmol), **4** (115 mg, 0.68 mmol), solvent free reaction. ^a^ Measured by GC/MS; ^b^ isolated yield in parenthesis.

**Table 4:** Recycling results of **A**-catalyzed phospha-Michael addition of dimethyl phosphite (**1b**) with 2-benzylidenemalononitrile (**3**).

Cycle No.	Time (h)	Temp. (°C)	Yield (%) ^a^	TON
**1**	1	80	100 (96) ^b^	20
**2**	1	80	99	19.8
**3**	1	80	99 (95)	19.8
**4**	1	80	97	19.4
**5**	1	80	99 (94)	19.8
**6**	1.5	80	95	19
**7**	1.5	80	96	19.2
**8**	2	80	98	19.6

Reaction conditions: T = 80 °C, cat. **A** (5 mol%), **1b** (0.14 mL, 1.5 mmol), **3** (115 mg, 0.75 mmol), solvent free reaction. ^a^ Measured by GC/MS; ^b^ isolated yield in parenthesis.

**Table 5:** Recycling results of **A**-catalyzed phospha-Michael addition of dimethyl phosphite (**1b**) with 2-(4-methylbenzylidene)malononitrile (**4**).

Cycle No.	Time (h)	Temp. (°C)	Yield (%) ^a^	TON
**1**	2	80	100	20
**2**	2	80	100 (96) ^b^	20
**3**	2	80	100 (96)	20
**4**	2	80	100 (97)	20
**5**	2	80	100	20
**6**	2	80	100	20
**7**	2	100	100	20
**8**	4	100	90	18

Reaction conditions: T = 80–100 °C, cat. **A** (5 mol%), **1b** (0.12 mL, 1.37 mmol), **4** (115 mg, 0.68 mmol), solvent free reaction. ^a^ Measured by GC/MS; ^b^ isolated yield in parenthesis.

**Table 6:** Recycling results of **A**-catalyzed phospha-Michael addition of triethyl phosphite (**2**) 2-benzylidenemalononitrile (**3**).

Cycle No.	Time (h)	Temp. (°C)	Yield (%) ^a^	TON
**1**	1	80	100 (96) ^b^	20
**2**	1	80	100	20
**3**	1	80	100 (97)	20
**4**	1	80	100	20
**5**	2	80	100 (97)	20
**6**	2	100	100	20
**7**	2	100	100	20
**8**	4	100	75	15

Reaction conditions: T = 80–100 °C, cat. **A** (5 mol%), **2** (0.25 mL, 1.5 mmol), **3** (115 mg, 0.75 mmol), solvent free reaction. ^a^ Measured by GC/MS; ^b^ isolated yield in parenthesis.

**Table 7:** Recycling results of **B**-catalyzed phospha-Michael addition of diisopropyl phosphite (**1**) with 2-benzylidenemalononitrile (**3**) at 80 °C.

Cycle No.	Time (h)	Temp. (°C)	Yield (%) ^a^	TON
**1**	1	80	100	20
**2**	1	80	100	20
**3**	1	80	100	20
**4**	1	80	100	20
**5**	1	80	99	19.8
**6**	1	80	98	19.6
**7**	1	80	98	19.6
**8**	1	80	96	19.2

Reaction conditions: T = 80 °C, cat. **B** (5 mol%), **1a** (0.25 mL, 1.5 mmol), **3** (115 mg, 0.75 mmol), solvent free reaction. ^a^ Measured by GC/MS.

**Table 8:** Recycling results of **B**-catalyzed phospha-Michael addition of diisopropyl phosphite (**1a**) with 2-(4-methylbenzylidene)malononitrile (**4**).

Cycle No.	Time (h)	Temp. (°C)	Yield (%) ^a^	TON
**1**	4.5	80	98 (98) ^b^	19.6
**2**	4.5	80	98	19.6
**3**	4.5	80	98 (96)	19.6
**4**	4.5	80	97	19.4
**5**	4.5	80	97 (95)	19.4
**6**	4.5	80	96	19.2
**7**	4.5	80	94	18.8
**8**	4.5	80	94	18.8

Reaction conditions: T = 80 °C, cat. **B**, 4-23F-py, (5 mol%), **1a** (0.23 mL, 1.37 mmol), **4** (115 mg, 0.68 mmol), solvent free reaction. ^a^ Measured by GC/MS; ^b^ isolated yield in parenthesis.

**Table 9:** Recycling results of **B**-catalyzed phospha-Michael addition of dimethyl phosphite (**1b**) with 2-benzylidenemalononitrile (**3**).

Cycle No.	Time(h)	Temp. (°C)	Yield (%) ^a^	TON
**1**	1	80	90 (88) ^b^	18
**2**	1	80	87	18.4
**3**	1	80	90 (87)	18
**4**	1	90	90	18
**5**	1	90	85 (84)	17
**6**	1	90	85	17
**7**	1.5	90	90	18
**8**	3	90	83	16.6

Reaction conditions: T = 80–90 °C, cat. **B** (5 mol%), **1b** (0.14 mL, 1.5 mmol), **3** (115 mg, 0.75 mmol), solvent free reaction. ^a^ Measured by GC/MS; ^b^ isolated yield in parenthesis.

## Data Availability

The data presented in this study are available in the article.

## Supplementary Materials

The following are available online: kinetic studies, GC/MS data of reactants and products and ^1^H NMR spectra of products.

## Sample Availability

Catalyst **A** and some of the fluorous compounds are available from the authors.
