# Supplementary material for: Recoverable Phospha-Michael Additions Catalyzed by a 4-N,N-Dimethylaminopyridinium Saccharinate Salt or a Fluorous Long-Chained Pyridine: Two Types of Reusable Base Catalysts
Source: Molecules. 2021 Feb 22;26(4):1159. doi: 10.3390/molecules26041159 (PMC7926848; doi:10.3390/molecules26041159)
Supplement: Supplementary file 1 [file molecules-26-01159-s001.pdf]

Recoverable phospha-Michael additions catalyzed by a 4-N,N-dimethylaminopyridinium saccharinate salt and a fluorous long-chained pyridine: Two types of reusable base catalysts

Eskedar Tessema [1, †], Vijayanath Elakkat [1,†], Chiao-Fan Chiu [2,3,*], Jing-Hung Zheng [1], Ka Long Chan [1], Chia-Rui Shen[4,5], Peng Zhang[6,*] and Norman Lu[1,7,*]

1. Institute of Organic and Polymeric Materials, National Taipei University of Technology, Taipei 106, Taiwan; eskedartessema18@gmail.com (E.T.); vijayanathe123@gmail.com (V.E.); st121321@gmail.com (J-H.Z.); kalongabc@gmail.com (K.L.C.)
2. Department of Pediatrics, Linkou Medical Center, Chang Gung Memorial Hospital, Taoyuan 333, Taiwan; chioufan2008@gmail.com
3. Graduate Institute of Clinical Medical Sciences, College of Medicine, Chang Gung University, Taoyuan 333, Taiwan
4. Department of Medical Biotechnology and Laboratory Sciences, College of Medicine, Chang Gung Univer-sity, Taoyuan 333, Taiwan; crshen@mail.cgu.edu.tw
5. Department of Ophthalmology, Linkou Medical Center, Chang Gung Memorial Hospital, Taoyuan 333, Taiwan
6. Department of Chemistry, University of Cincinnati, Cincinnati, Ohio 45221-0172, United States; peng.zhang@uc.edu (P.Z.)
7. Development Center for Smart Textile, National Taipei University of Technology, Taipei 106, Taiwan.

Supplementary Materials (SM)

1. General Procedures S2
2. GC/MS analysis S2
3. Reaction mechanism of base assisted phospha-Michael addition S3
4. Kinetic Studies S3
  4.1. Kinetic Studies of Catalyst A
  4.2. Kinetic Studies of Catalyst B
5. FT-IR Spectra of catalysts S5
  5.1. FT-IR Spectra of catalyst A
  5.2. FT-IR Spectra of catalyst B
6. Identification of phospha-Michael addition products S6
  6.1. Identification data of products
  6.1.1. GC/MS data of reactants and products
    6.1.1.1. GC/MS data of reactants
    6.1.1.2. GC/MS data of products
  6.1.2. ^{1}H NMR spectra of products

6. References S15

1. General procedure

General Procedures HP 6890 GC containing a 30 m 0.250 mm HP-1 capillary column with a 0.25 mm stationary phase film thickness was used to censor the reaction. The same GC instrument with a 5973 series mass selective detector was used to Acquire GC/MS data. The flow rate was 1 mL/min and splitless. Infra-red spectra were obtained on a Perkin Elmer RX I FT-IR Spectrometer. NMR spectra were recorded on Bruker AM 500, AM300 and Joel AM 200 using 5 mm sample tubes. CD_3OD, $CDCl_3$, deuterated DMF and deuterated DMSO were the references for both ^{1}H- and ^{13}C-NMR spectra; and Freon® 11 ($CFCl_3$) was the reference for ^{19}F NMR spectra.

2. GC/MS analysis

The GC/MS samples were prepared by taking 2 drops of the product mixtures from the solution after being treated with 5 mL of non-polar solvent and followed by recovering the catalyst. Then 2 drops of sample was dissolved in 1 mL of dichloromethane (CH_2Cl_2); and then 1 mL of DI water was added for extracting the water-soluble materials. After the mixture was well mixed, the 2 μL of the sample was taken by using micro syringe from the CH_2Cl_2 layer for GC/MS injection.

3. Reaction mechanism of base-catalyzed phospha-Michael addition reaction

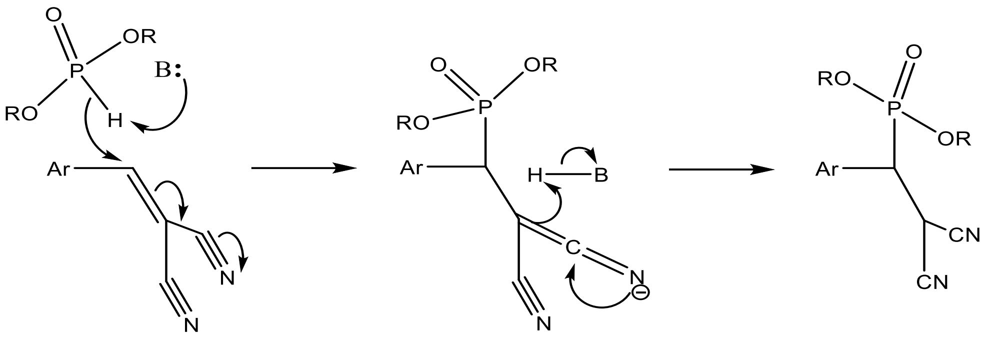

Figure S1. Reaction mechanism of base-catalyzed phospha-Michael addition of **1a** or **1b** with **3** or **4. [Note:** B= base] [2,3].

4. Kinetic Studies

4.1. Kinetic studies of catalyst A

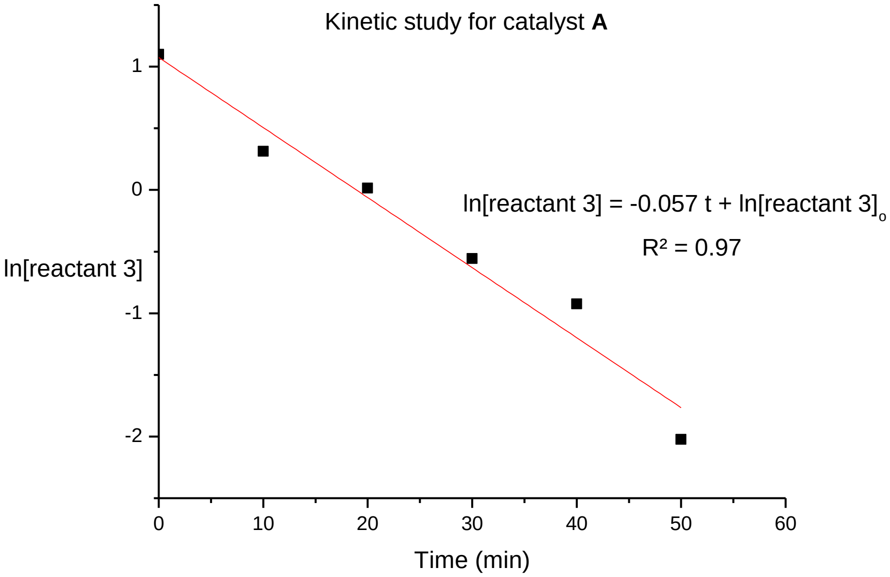

Time (min)	ln [reactant **3**]
0	1.099
10	0.313
20	0.015
30	-0.556
40	-0.924
50	-2.022

Figure S2. The linear plot of **A**-catalyzed phospha-Michael addition of benzylidenemalononitrile (**3**) at 80 °C. (Note: t= time, $[\text{reactant } \mathbf{3}]_o$= 3 M)

4.2. Kinetic studies of catalyst B

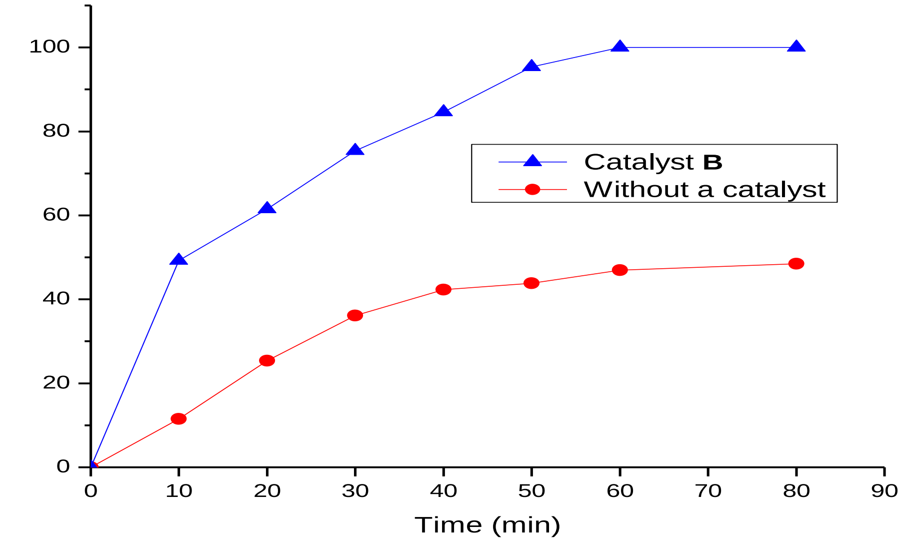

Figure S3. Kinetic study of phospha-Michael addition reactionof diisopropyl phosphite (**1a**) with benzylidenemalononitrile (**3**) with and without a catalyst **B**. [4,5].

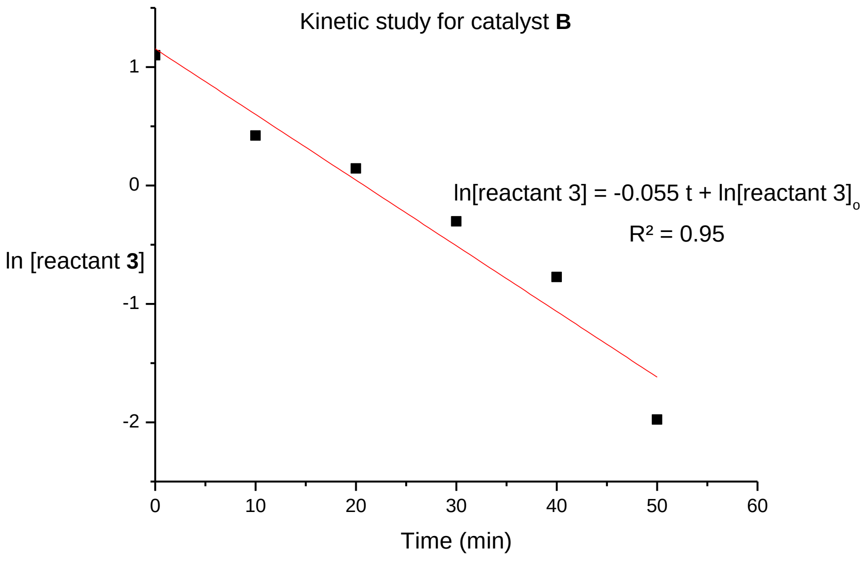

Time (min)	ln [reactant **3**]
0	1.099
10	0.421
20	0.143
30	-0.303
40	-0.773
50	-1.977

Figure S4. The linear plot of **B**-catalyzed phospha-Michael addition of benzylidenemalononitrile (**3**) at 80 °C. (Note: t= time, [reactant **3**]$_o$=3 M)

5. FT-IR Spectra of catalysts A and B

5.1. FT-IR spectrum of catalyst A

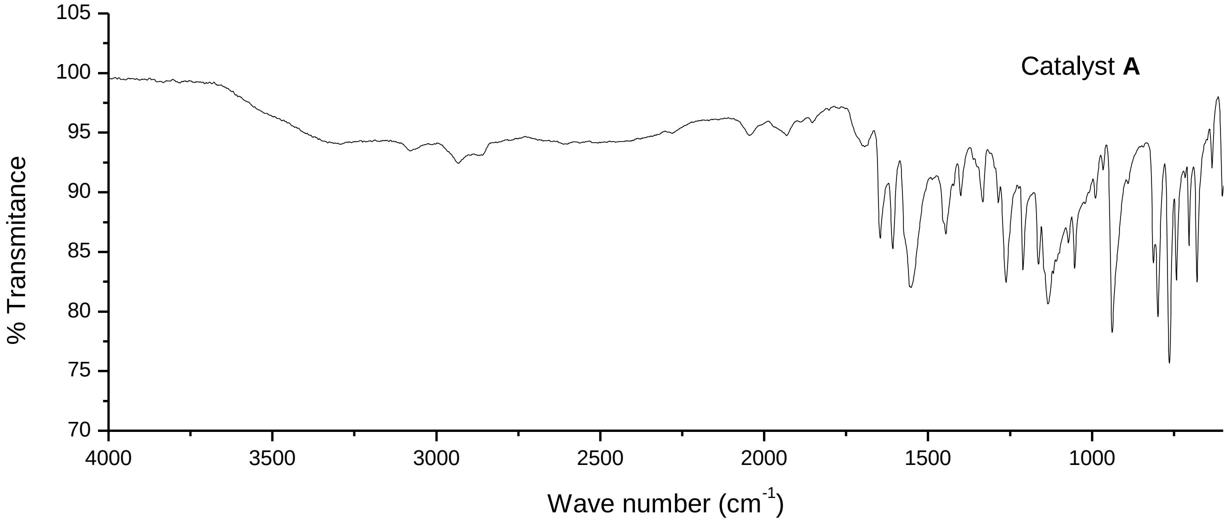

Figure S5. FT-IR spectrum of catalyst DMAP·HSac (**A**). [Note: The C=O stretching frequency can be observed at 1643 cm^{-1}, R-SO_2-N vibrational frequency can be observed at 1333, 1210 and 1136 cm^{-1}].

5.2. FT-IR spectrum of catalyst B

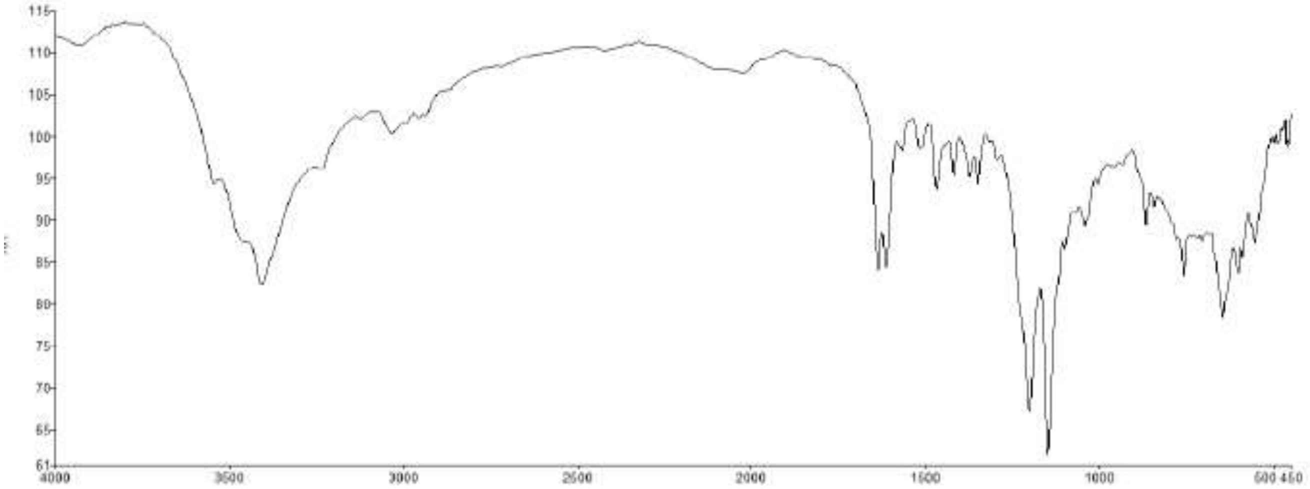

Figure S6. FT-IR spectrum of catalyst 4-23F-py (**B**). [Note: The C-F stretching frequency can be observed at 1200 and 1146. cm^{-1}].

6. Identification of phospha-Michael addition products and related compounds

Most of the **A** or **B**-catalyzed phospha-Michael addition products from Tables 1-9 are known compounds which exhibited spectroscopic data identical to those reported in the literature [1]. All of the products have been checked by GC/MS and NMR methods.

6.1. Identification data of reactants and products

6.1.1. GC/MS data of reactants and products

6.1.1.1. GC/MS data of reactants

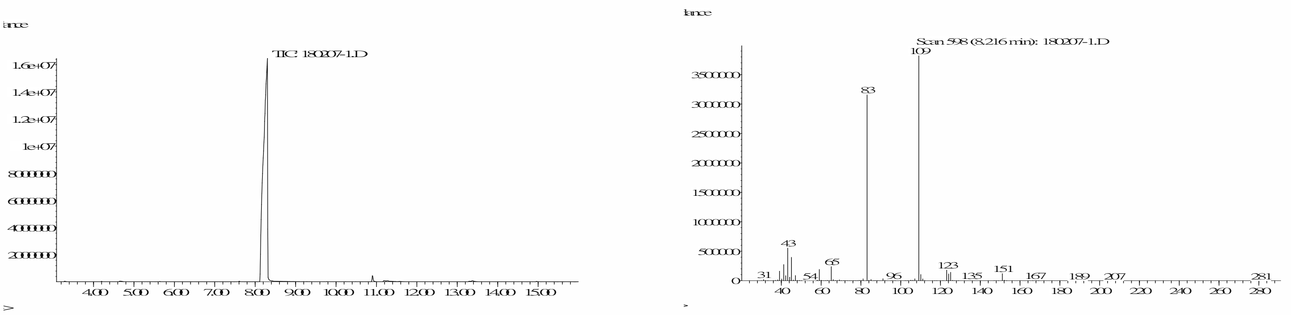

Figure S7. GC/MS data [(L): GC; (R): mass] of diisopropyl phosphite (**1a**). GC/MS (M^+; m/z=):

166 (M^+), 151 (M^+-O), 123 ($C_3H_8O_3P$), 109 ($C_3H_8O_2P$), 59 (C_3H_7O), 43 (C_3H_7).

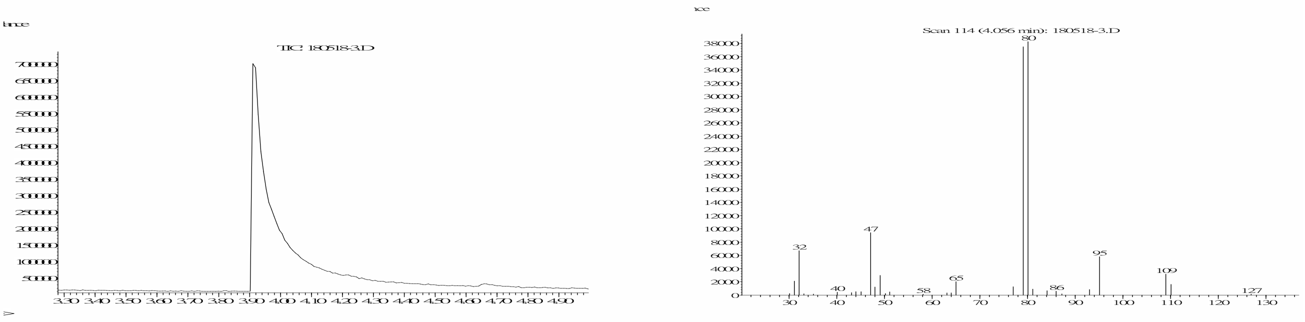

Figure S8. GC/MS data [(L): GC; (R): mass] of dimethyl phosphite (**1b**). GC/MS (M^+; m/z=):

110 (M^+), 95 (M^+-CH_3), 80 (HO_3P), 47 (OP).

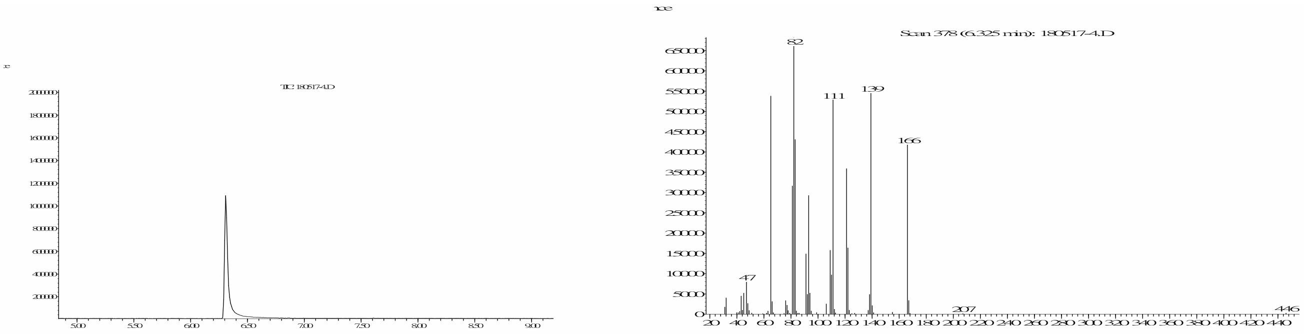

Figure S9. GC/MS data [(L): GC; (R): mass] of triethyl phosphite (**2**). GC/MS (M^+; m/z=):166 (M^+), 139 (M^+-CH_2CH_3), 111 (M^+-2(CH_2CH_3)), 47 (OP).

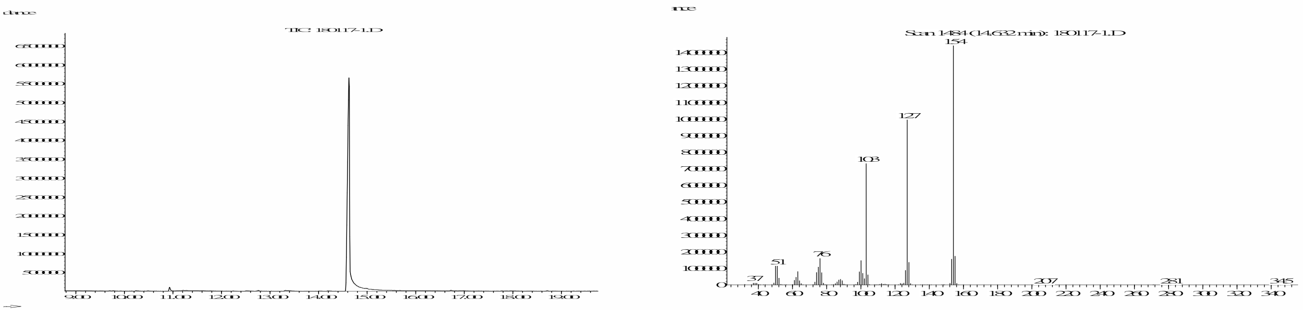

Figure S10. GC/MS data [(L): GC; (R): mass] of 2-benzylidenemalononitrile (**3**). GC/MS (M^+; m/z=): 154 (M^+), 127 (M^+-CN), 76 (C_6H_5), 53 (C_3HN).

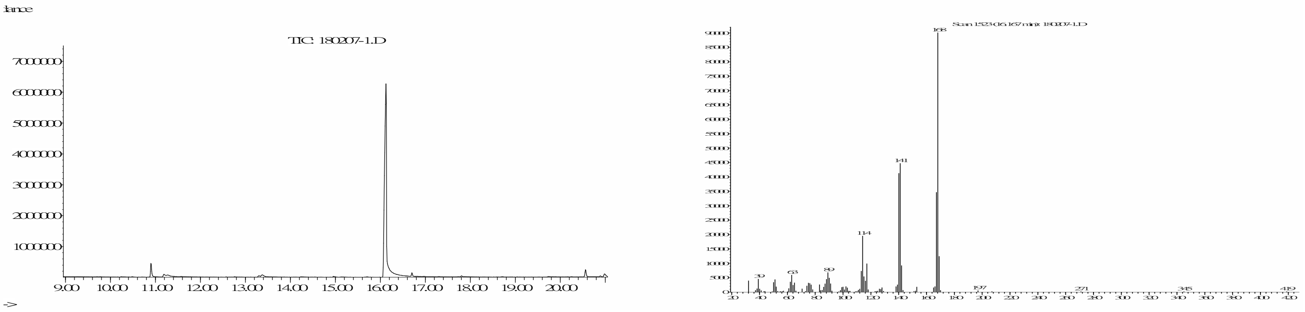

Figure S11. GC/MS data [(L): GC; (R): mass] of 2-(4-methylbenzylidene)malononitrile (**4**). GC/MS (M^+; m/z=): 168 (M^+), 141 ($C_{10}H_9N$), 114 (C_9H_6), 89 (C_7H_5).

6.1.1.2. GC/MS data of products [Note: reactants (**3** &**4**) are the limiting reagents showing up at 14.6 and 16.1 mins, respectively. Thus, for this type of clean reactions, the total disappearance of reactant peak means the complete conversion of the reaction (100% yield).

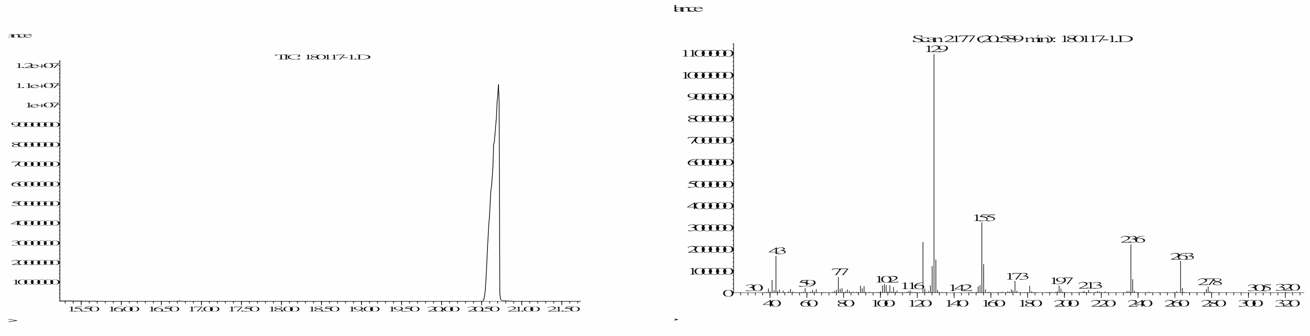

Figure S12. GC/MS data [(L): GC; (R): mass] of [1-(phenyl)-2,2-dicyanoethyl] phosphonic acid diisopropyl ester. GC/MS (M^+; m/z=): 320 (M^+), 278 (M^+-C_3H_7), 263 ($C_{13}H_{14}N_2O_2P$), 236 ($C_{10}H_7N_2O_3P$), 155 ($C_{10}H_7N_2$).

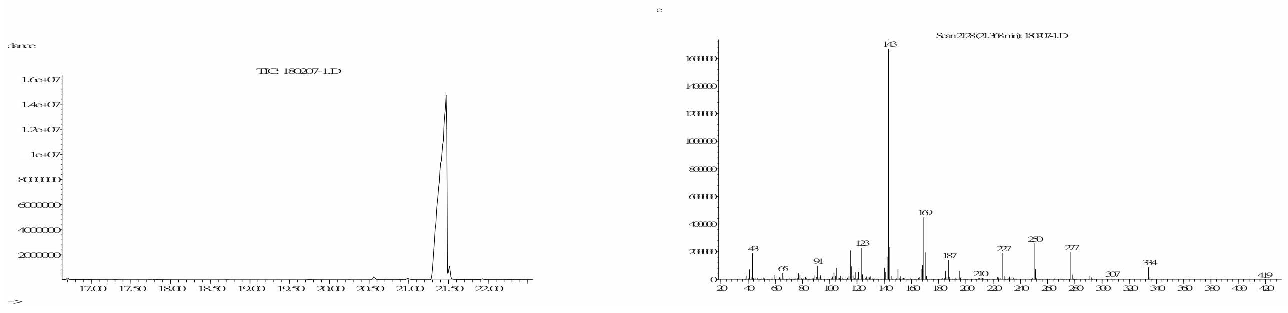

Figure S13. GC/MS data [(L): GC; (R): mass] of [1-(tolyl)-2,2-dicyanoethyl] phosphonic acid diisopropyl ester. GC-MS (M^+; m/z=): 334 (M^+), 277 (M^+-C_3H_6), 250 ($C_{12}H_{13}N_2O_2P$), 169 ($C_{11}H_9N_2$), 143 (C_{10}H7N).

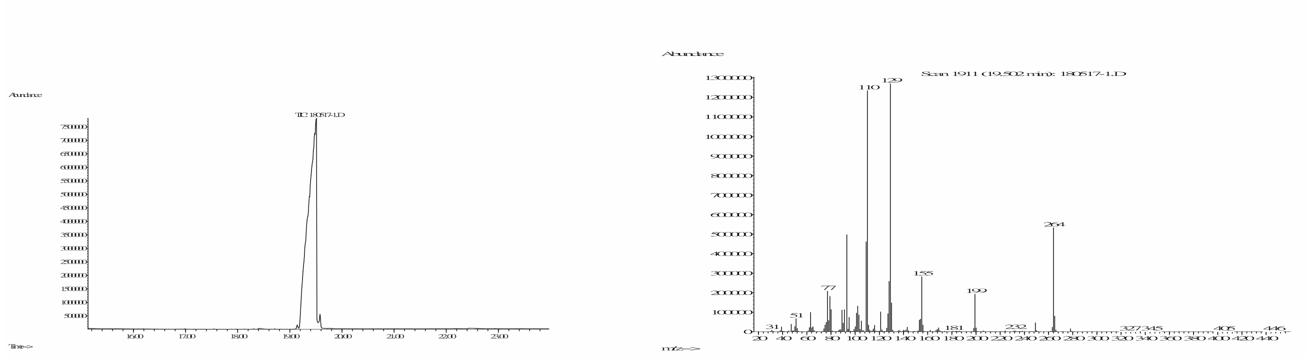

Figure S14. GC/MS data [(L): GC; (R): mass] of [1-(phenyl)-2,2-dicyanoethyl] phosphonic acid dimethyl ester. GC-MS (M^+; m/z=): 264 (M^+), 155 ($C_{10}H_7N_2$), 129 (C_9H_7N), 110 (C_9H_2).

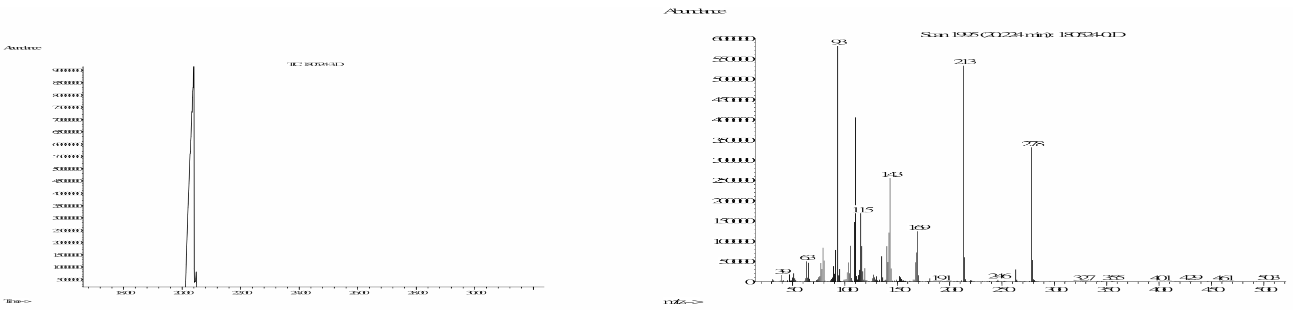

Figure S15. GC/MS data [(L): GC; (R): mass] of [1-(tolyl)-2,2-dicyanoethyl] phosphonic acid dimethyl ester. GC-MS (M^+; m/z=): 278 (M^+), 213 ($C_{10}H_{14}O_3P$), 169 ($C_{11}H_9N_2$), 93 ($C_2H_6O_2P$).

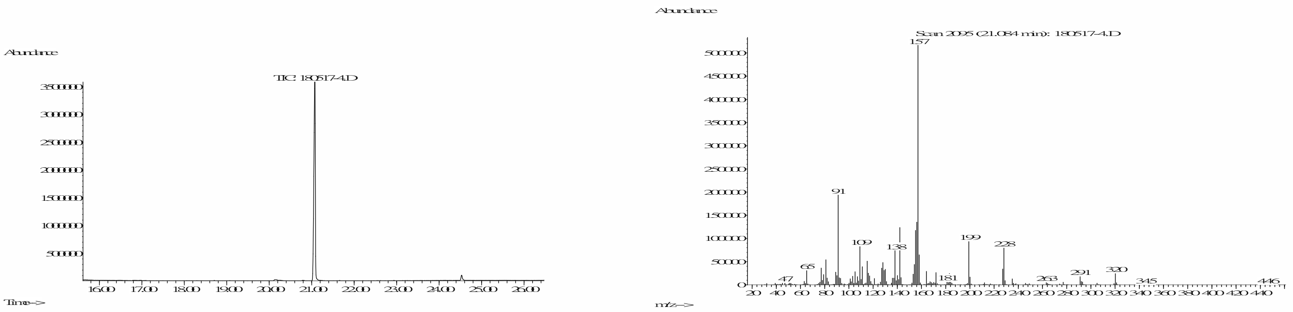

Figure S16. GC/MS data [(L): GC; (R): mass] of [1-(phenyl)-2,2-dicyanobutyl] phosphonic acid diethyl ester. GC-MS (M^+; m/z=): 320 (M^+), 199 ($C_{10}H_4N_2OP$), 157 (C_9H_4NP).

6.1.2 ^{1}H NMR spectra of products

A. [1-(phenyl)-2,2-dicyanoethyl] phosphonic acid diisopropyl ester

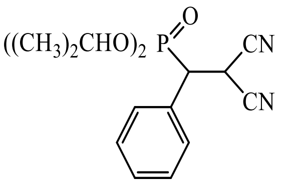

(product from Tables 1, 2 and 7)

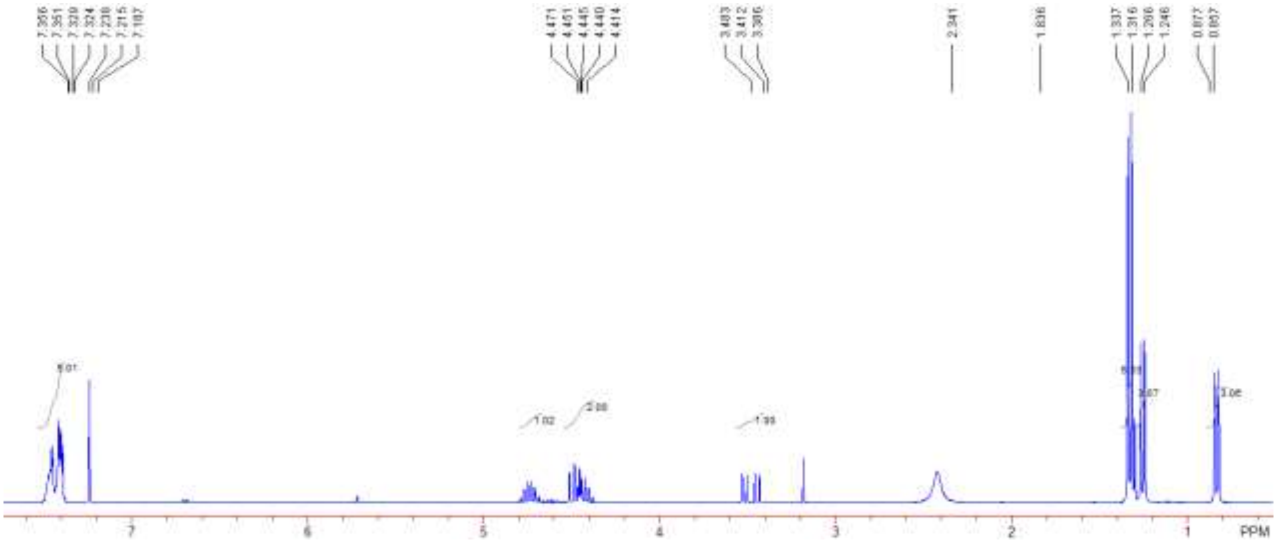

Figure S17. ^{1}H NMR spectrum of [1-(phenyl)-2,2-dicyanoethyl] phosphonic acid diisopropyl ester.

[1-(phenyl)-2,2-dicyanoethyl] phosphonic acid diisopropyl ester. ^{1}H NMR (300 MHz, $CDCl_3$-d_6) δ (ppm)= 7.32 ~7.40 (5H, m, Ar-H), 4.67~4.80 (1H, m, -CH$(CN)_2$), 4.40~4.50 (2H, m, -OCH), 3.44 (1H, dd, $^{3}J_{HH}$ =8 Hz, $^{2}J_{HP}$=21 Hz, CHP), 1.33 (6H, d, $^{3}J_{HH}$= 6 Hz, -CHC$\underline{H}_3$), 1.06 (6H, dd, $^{3}J_{HH}$= 6 Hz, $^{4}J_{HP}$= 117 Hz, -CHC$\underline{H}_3$).

B. [1-(tolyl)-2,2-dicyanoethyl] phosphonic acid diisopropyl ester

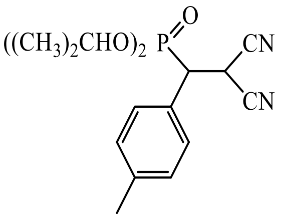

(product from Tables 3 and 8)

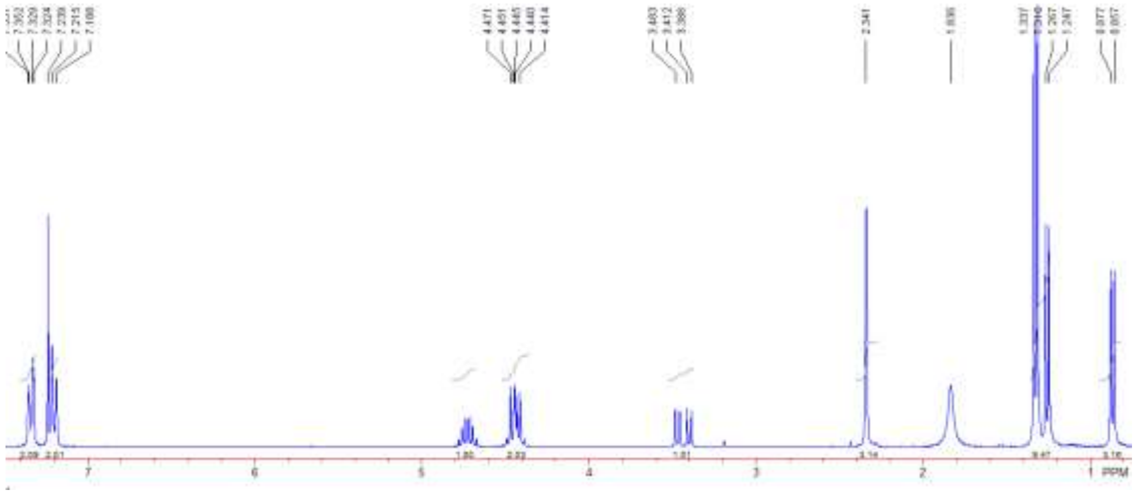

Figure S18. ^{1}H NMR spectrum of [1-(tolyl)-2,2-dicyanoethyl] phosphonic acid diisopropyl ester.

[1-(tolyl)-2,2-dicyanoethyl] phosphonic acid diisopropyl ester. ^{1}H NMR (300 MHz, $CDCl_3$-d_6) δ (ppm) = 7.34 (2H, d, $^{3}J_{HH}$= 8 Hz, H-2, H-6), 7.21 (2H, d, $^{3}J_{HH}$= 8 Hz, H-3, H-5), 4.67~4.78 (1H, m, -CH(CN)$_2$), 4.39~4.49 (2H, m, -OCH), 3.44 (1H, dd, $^{3}J_{HH}$= 8 Hz, $^{2}J_{HP}$= 21 Hz, CHP), 2.34 (1H, s, Ar-CH_3), 1.33 (6H, d, $^{3}J_{HH}$= 6 Hz, -CHC$\underline{H}_3$), 1.06 (6H, dd, $^{3}J_{HH}$= 6 Hz, $^{4}J_{HP}$=117 Hz, -CHC$\underline{H}_3$).

C. [1-(phenyl)-2,2-dicyanoethyl] phosphonic acid dimethyl ester

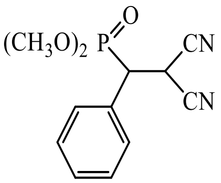

(product from Tables 4 and 9)

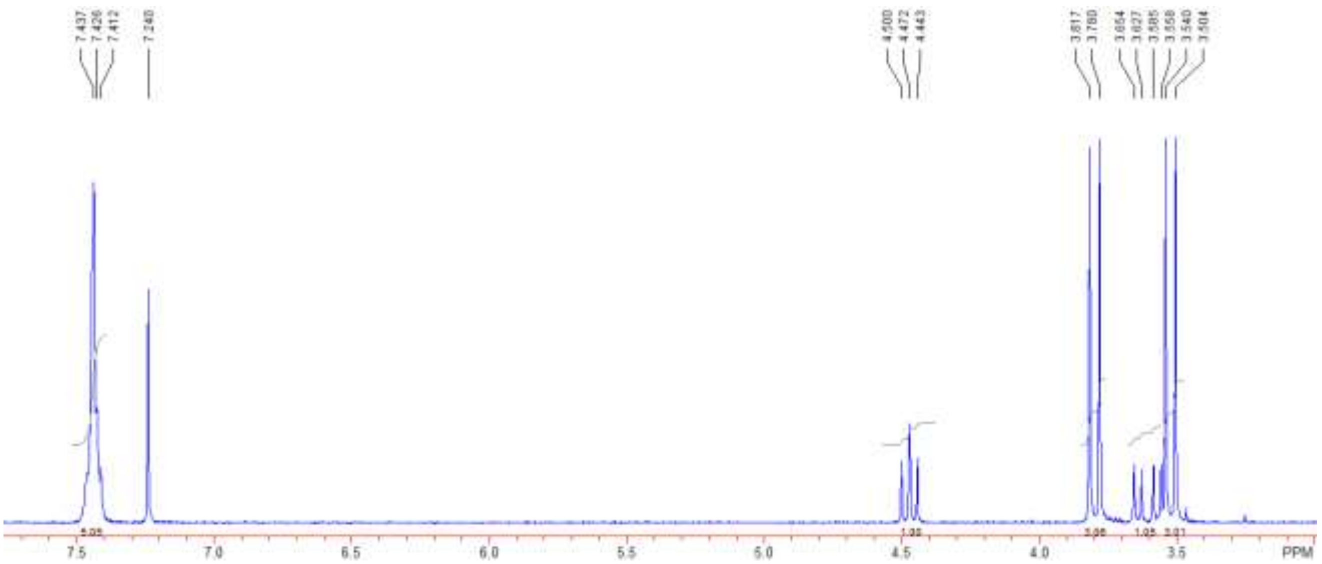

Figure S19. ^{1}H NMR spectrum of [1-(phenyl)-2,2-dicyanoethyl] phosphonic acid dimethyl ester.

[1-(phenyl)-2,2-dicyanoethyl] phosphonic acid dimethyl ester. ^{1}HNMR (300 MHz, $CDCl_3$-d_6) δ (ppm) = 7.41~7.46 (5H, m, Ar-H), 4.47 (1H, t, $^{3}J_{HH}$ = 8.4 Hz, -CH(CN)$_2$), 3.80 (3H, d, $^{3}J_{HP}$= 11 Hz, OCH_3), 3.60 (1H, dd, $^{3}J_{HH}$= 9 Hz, $^{2}J_{HP}$= 21 Hz, CHP), 3.52 (3H, d, $^{3}J_{HP}$= 11 Hz, -OCH_3).

D. [1-(tolyl)-2,2-dicyanoethyl] phosphonic acid dimethyl ester

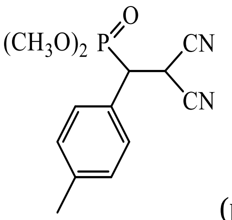

(product from Table 5)

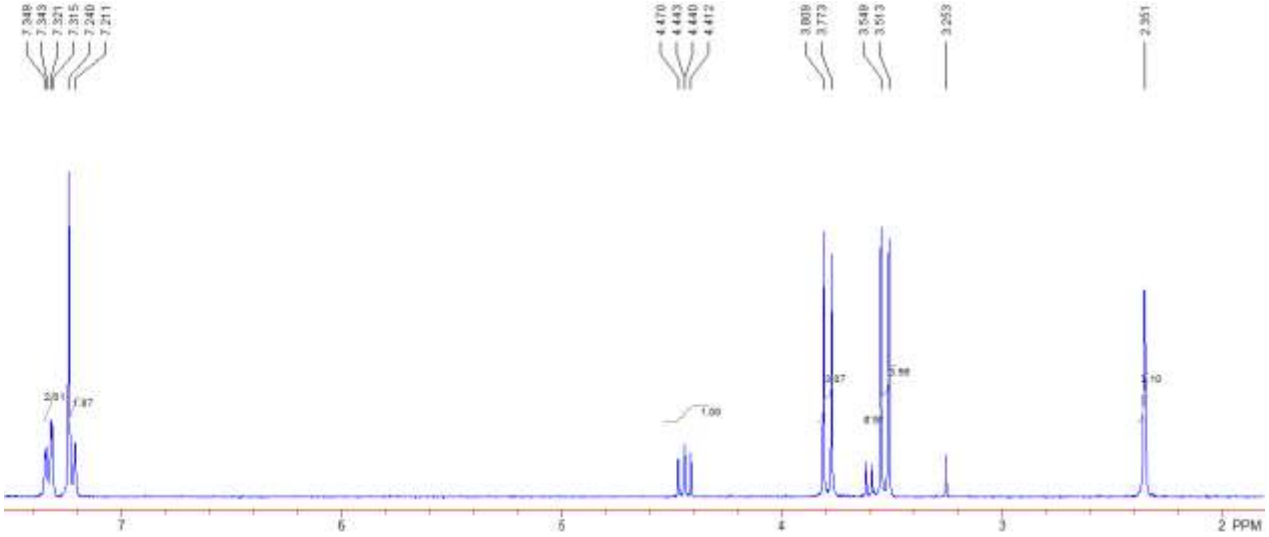

Figure S20. ^{1}H NMR spectrum of [1-(tolyl)-2,2-dicyanoethyl] phosphonic acid dimethyl ester.

[1-(tolyl)-2,2-dicyanoethyl] phosphonic acid dimethyl ester. ^{1}HNMR (300 MHz, $CDCl_3$-d_6) δ (ppm) = 7.34 (2H, d, $^{3}J_{HH}$= 8 Hz, H-2, H-6), 7.23 (2H, d, $^{3}J_{HH}$= 8 Hz, H-3, H-5), 4.44 (1H, t, $^{3}J_{HH}$= 8.4 Hz, CHP), 3.79 (3H, d, $^{3}J_{HP}$=10.8 Hz, $-OCH_3$), 3.50~3.62 (4H, m, $-OCH_3$, $-CH(CN)_2$), 2.35 (3H, s, Ar-CH_3).

E. [1-(phenyl)-2,2-dicyanobutyl] phosphonic acid diethyl ester

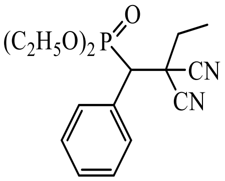

(product from Table 6)

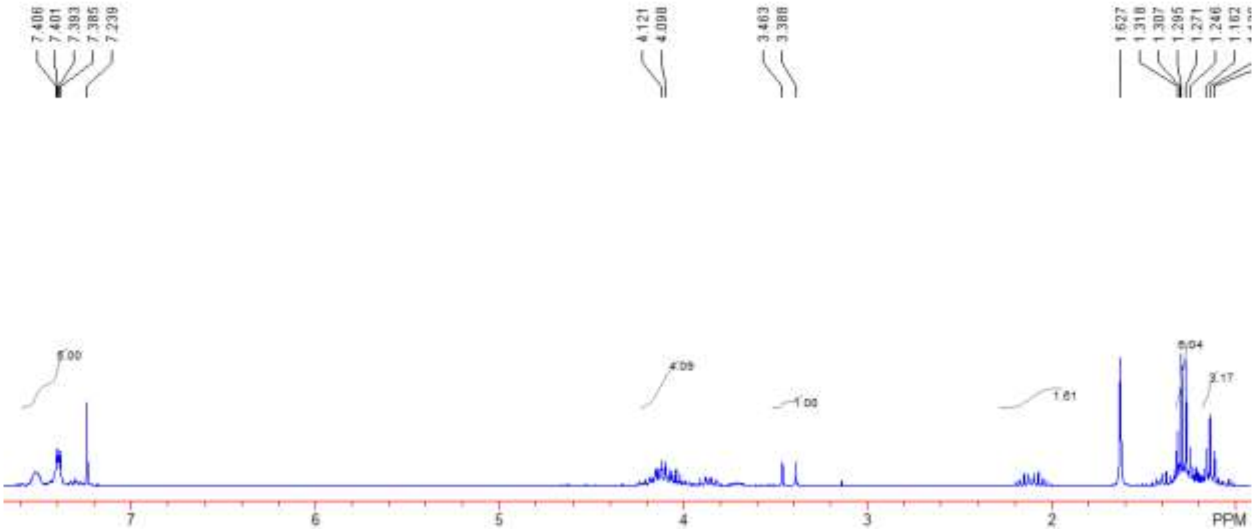

Figure S21. ^{1}H NMR spectrum of [1-(phenyl)-2,2-dicyanobutyl] phosphonic acid diethyl ester.

[1-(phenyl)-2,2-dicyanobutyl] phosphonic acid diethyl ester. ^{1}H-NMR (300 MHz, $CDCl_3$-d_6) δ (ppm) = 7.38~7.52 (5H, m, Ar-H), 4.04~4.15 (4H, m, $POCH_2$), 3.43 (1H, d, $^2J_{HP}$= 22 Hz, CHP), 2.07~2.15 (2H, m, $CH_2C(CN)_2$), 1.2~1.4 (6H, m, $POCH_2C\underline{H}_3$), 1.14 (3H, t, $^3J_{HH}$= 7 Hz, $C\underline{H}_3CH_2C(CN)_2$).

7. References

1. Hosseini-Sarvari, M.; Etemad, S., Nanosized zinc oxide as a catalyst for the rapid and green synthesis of β-phosphono malonates. *Tetrahedron* **2008**, *64(23)*, 5519-5523.
2. Lenz, J.; Pospiech, D.; Komber, H.; Paven, M.; Albach, R.; Mentizi, S.; Langstein, G.; Voit, B., Synthesis of the H-phosphonate dibenzo [d, f] [1, 3, 2] dioxaphosphepine 6-oxide and the phospha-Michael addition to unsaturated compounds. *Tetrahedron* ***2019***, *75,* 1306-1310.
3. Rulev, A. Y., Recent advances in Michael addition of H-phosphonates. *RSC Advances* **2014**, *4,* 26002-26012.
4. Hosseini-Sarvari, M.; Etemad, S. Nanosized zinc oxide as a catalyst for the rapid and green synthesis of β-phosphono malonates. *Tetrahedron* **2008**, *64*, 5519-5523.
5. Sobhani, S.; Bazrafshan, M.; Delluei, A.A.; Parizi, Z.P. Phospha-michael addition of diethyl phosphite to α, β-unsaturated malonates catalyzed by nano γ-Fe_2O_3-pyridine based catalyst as a new magnetically recyclable heterogeneous organic base. *Applied Catalysis A: General* **2013**, *454*, 145-151.
